# Association between alcohol intake and measures of incident CKD: An analysis of nationwide health screening data

**DOI:** 10.1371/journal.pone.0222123

**Published:** 2019-09-20

**Authors:** Minseon Park, Seung Min Lee, Hyung-Jin Yoon

**Affiliations:** 1 Department of Family Medicine, Seoul National University Hospital, Seoul, Republic of Korea; 2 Department of Biomedical Engineering, Seoul National University College of Medicine, Seoul, Republic of Korea; Beijing Key Laboratory of Diabetes Prevention and Research, CHINA

## Abstract

To evaluate the association between alcohol intake and incident chronic kidney disease measures as well as the sex differences in this association, we analyzed health screening data of 14,190,878 adults who underwent health screening ≥3 times and had glomerular filtration rate (eGFR) ≥60 mL/min/1.73 m^2^ and normal proteinuria at baseline. eGFR was calculated with the Chronic Kidney Disease Epidemiology Collaboration equation. Proteinuria was defined as ≥1+ dipstick proteinuria and low eGFR as <60 mL/min/1.73 m^2^. The risk of incident proteinuria and low eGFR was analyzed with an extended Cox model with alcohol intake level as a time-varying determinant and the annual change of eGFR with generalized linear model. A J-shape association of alcohol intake with the incident proteinuria was observed in men (adjusted hazard ratio [aHR], 0.961, 95% confidence interval [CI], 0.953–0.970 in men drinking alcohol <10 g/day; aHR 1.139, 95% CI, 1.123–1.154 in men drinking alcohol ≥40 g/day, compared with non-drinking men), and a positive association was seen in women (aHR, 1.034, 95% CI, 1.023–1.044 in women drinking alcohol <10 g/day; aHR, 1.094, 95% CI, 1.034–1.158 in women drinking alcohol ≥40 g/day, compared with non-drinking women). In both sexes, an inverse association of alcohol intake with the annual eGFR decline and incident low eGFR was observed. This study observed a beneficial effect of moderate alcohol intake on incident proteinuria in men and a protective effect of alcohol intake of any amount on the annual eGFR decline and incident low eGFR in both sexes. The long-term implications of these observations need to be elucidated with future studies.

## Introduction

Although excess intake of alcohol has been associated with various medical conditions, beneficial effects of moderate alcohol intake on several diseases such as cardiovascular or cerebrovascular disorders have been reported [1].

Chronic kidney disease (CKD), of which current international definition is decreased kidney function shown by glomerular filtration rate (GFR) of less than 60 mL/min per 1.73 m^2^ or markers of kidney damage frequently shown by albuminuria or proteinuria [2], is one of the most important global health issues because of its heavy socio-economic burden, and the cost-effectiveness of CKD prevention programs in high-risk subjects has been proven [3]. Therefore, the influences of various lifestyles on the development of CKD should be elucidated to develop effective strategies to prevent CKD. Considering the high percentage of the population who drink, the relationship between alcohol intake and CKD risk deserves much more attention.

Observations of the association between alcohol and incident CKD have been inconsistent, and inverse, positive, and J-shaped associations have been reported [4–13]. Although there may be many possible explanations for this inconsistency, such as small sample sizes, inconsistent definition of alcohol intake levels, and inconsistent definition of CKD, the lack of representativeness of the samples participating in the previous studies due to small sample size or possible difference in lifestyles between those who were actively involved in cohort studies and those who were not, may be one of the reasons. Therefore, studies with sample populations that cover a substantial proportion of a whole population may provide a more reliable answer to this important research question.

Because moderate to heavy alcohol consumption has been associated with a higher health risk in women than in men [14], there may be a sex difference in the association between alcohol and CKD. The sex differences in this association have not been well studied, and the few existing studies have reported inconsistent results [15, 16].

To evaluate the association between alcohol intake, the risk of incident proteinuria, and change in estimated GFR (eGFR) as well as the possible sex differences in this association, we analyzed health screening data of more than 14 million out of 36 million Korean adults, who underwent health screening ≥3 times between 2009 and 2016 and who had a negative urine dipstick test for proteinuria and an eGFR ≥60 mL/min per 1.73 m^2^ at baseline.

## Methods

### Participants

In Korea, regular health screening at designated screening hospitals every year or once every 2 years is obligatory. More than 40% of the 36 million adult Koreans who were older than 20 years were enrolled, and the participation rate was higher than 66% [17]. Briefly, lifestyle factors, such as alcohol intake, exercise, smoking, past medical history, current medical conditions, and family history were collected using a nationally uniformed, structured questionnaire. Height, weight, and blood pressure were measured. Laboratory tests included blood hemoglobin, fasting serum glucose, liver function test, blood lipids, serum creatinine, urine dipstick for proteinuria, and chest x-ray.

**Fig 1:**
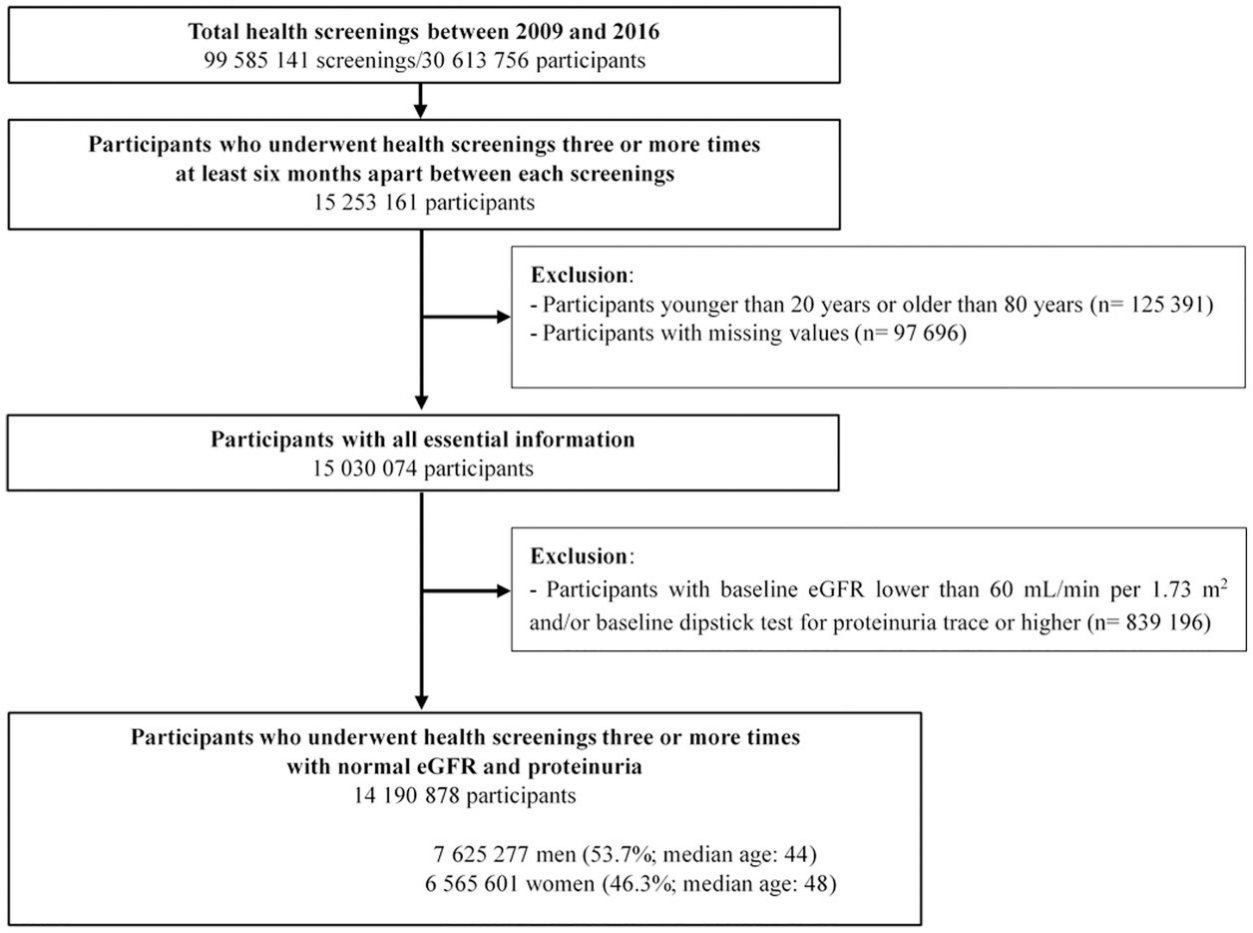
Selection of study participants.

Between 2009 and 2016, 99,585,141 health screenings were performed on 30,613,756 participants. Among 15,253,161 participants who underwent health screening ≥3 times during this period with at least 6 months apart between each screening, 125,391 participants were excluded due to baseline age <20 years or >80 years, 97,696 due to with missing values in the adjusting variables, and 839,196 due to with baseline eGFR <60 mL/min per 1.73 m^2^ and/or baseline dipstick proteinuria ≥trace. Finally, health screening data of 14,190,878 participants were analyzed (Fig 1).

The amount of alcohol intake was measured by the number of drinks per week, and one drink of all alcoholic beverages was calculated as 7.85 g of ethanol [18]. The average daily alcohol intake was categorized as no consumption, <10 g/day, 10 to 19.9 g/day, 20 to 39.9 g/day, and ≥40 g/day. Smoking status was categorized as nonsmoker, ex-smoker, and current smoker. Regular exercise was defined as ≥3 sessions per week of moderate-intensity exercise lasting >30 min and/or vigorous-intensity exercise for >20 min.

eGFR was calculated with the Chronic Kidney Disease Epidemiology Collaboration equation based on serum creatinine and adjusted as previously described [19]. Incident low eGFR was defined as a decrease in eGFR <60 mL/min per 1.73 m^2^ at any time during the follow-up period in the participants whose baseline eGFR was ≥60 mL/min per 1.73 m^2^. Incident proteinuria was defined as ≥1+ by spot urine dipstick test for proteinuria at any time during the follow-up period in the participants who showed negative dipstick test at baseline.

For subgroup analysis, the participants were subgrouped according to age (≤sex-specific median age vs >sex-specific median age; 44 years for men, 48 years for women), smoking status (non-smoker vs ex-smoker vs current smoker), and body mass index (BMI; ≤25 kg/m^2^ vs >25 kg/m^2^). For sensitivity analysis, the incident proteinuria was defined as urine dipstick test ≥2+ and incident low eGFR was defined as <55 mL/min per 1.73 m^2^

### Statistical analysis

The risk of incident proteinuria according to the level of daily alcohol intake was evaluated with an extended Cox model with the drinking status as a time-varying determinant, adjusted for the baseline characteristics. The adjust variables were age, sex, BMI, systolic blood pressure, fasting serum glucose, serum high-density lipoprotein cholesterol and triglycerides levels, eGFR, history of diabetes and hypertension medication, regular exercise, and smoking status.

The annual decline in eGFR according to the level of daily alcohol intake was estimated with a generalized linear model adjusted for baseline characteristics. The adjust variables were age, sex, BMI, systolic blood pressure, fasting serum glucose, serum high-density lipoprotein cholesterol and triglycerides levels, history of diabetes and hypertension medication, regular exercise, and smoking status. The risk of incident low eGFR according to the level of daily alcohol intake was analyzed with an extended Cox model with the drinking status as a time-varying determinant, adjusted for the same baseline characteristics. All statistical analyses were conducted using R 3.4.3 (http://www.R-project.org) and SAS version 9.4 (SAS Institute, Cary, NC).

The Institutional Review Board of Seoul National University Hospital waived the informed consents and approval because of the nature of this study, which retrospectively analyzed the national registry data.

## Results

The baseline characteristics of the participants are summarized in Table 1, respectively. Among men, 31.1% did not drink, 27.7% drank <10 g/day, 18.0% drank between 10 and 19.9 g/day, 16.3% drank between 20 and 39.9 g/day, and 6.9% drank ≥40 g/day. Among women, 74.2% did not drink, 19.7% drank <10 g/day, 3.8% drank between 10 and 19.9 g/day, 1.8% drank between 20 and 39.9 g/day, and 0.5% drank ≥40 g/day. Men who drank more, the older those were, and women who drank more, the younger those were (*p* for trends <0.001, both). In both men and women, the more alcohol they drank, the more weight they had and the higher the percentage of current smokers was (*p* for trends <0.001, all). The blood pressure of those who drank more alcohol tended to be higher compared with those who drank less in both sexes (*p* for trends <0.001, both). In both sexes, the serum level of high-density lipoprotein cholesterol and eGFR increased dose-dependently as the amount of alcohol ingested increased (*p* for trends <0.001, both; Table 1).

**Table 1:** General characteristics of the participants at baseline according to sex and drinking status.

	Men						Women					
	Average daily alcohol consumption						Average daily alcohol consumption					
	No	< 10 g	10–19.9 g	20–39.9 g	≥40 g	*P* for trend	No	< 10 g	10–19.9 g	20–39.9 g	≥40 g	*P* for tread
	2,371,899 (31.1%)	2,110,355 (27.7%)	1,375,498 (18.0%)	1,241,288 (16.3%)	526,237 (6.9%)		4,873,733 (74.2%)	1,292,163 (19.7%)	247,902 (3.8%)	120,548 (1.8%)	31,255 (0.5%)	
Age	47.7±14.1	42.9±12.9	43.0±12.3	43.5±11.9	45.2±12.7	<0.001	50.3±12.8	41.3±12.0	39.9±12.1	39.4±12.4	39.3±12.8	<0.001
Height (cm)	169.4±6.5	170.7±6.3	171.0±6.2	171.0±6.3	170.7±6.4	<0.001	156.3±5.9	158.4±5.7	158.8±5.7	159.0±5.8	159.2±5.9	<0.001
Weight (kg)	68.9±10.4	69.9±10.1	70.9±10.2	71.5±10.4	71.8±11.0	<0.001	56.9±8.2	56.5±8.1	57.2±8.5	57.7±8.8	58.3±9.3	<0.001
Smoking status												
Nonsmoker (%)	47.9%	32.6%	21.5%	16.7%	15.6%	<0.001	97.6%	93.3%	81.5%	71.1%	59.7%	<0.001
Ex-smoker (%)	22.2%	25.6%	26.5%	26.2%	25.5%	<0.001	0.8%	2.5%	5.4%	7.3%	8.5%	<0.001
Current Smoker (%)	30.0%	41.8%	52.0%	57.1%	58.9%	<0.001	1.6%	4.2%	13.1%	21.6%	31.9%	<0.001
Regular exercise (%)	31.5%	37.4%	38.1%	37.3%	34.6%	<0.001	26.8%	29.3%	29.4%	28.4%	27.7%	<0.001
History of DM medication (%)	5.0%	3.2%	3.3%	3.6%	4.6%	<0.001	4.4%	1.4%	1.3%	1.4%	1.7%	<0.001
History of HT medication (%)	11.5%	9.1%	10.3%	11.5%	13.3%	<0.001	15.4%	6.9%	7.3%	7.9%	8.5%	<0.001
Body mass index												
≤25 kg/m^2^	66.1%	66.9%	63.2%	60.4%	57.9%	<0.001	73.0%	80.4%	78.5%	77.1%	75.2%	<0.001
>25 kg/m^2^	33.9%	33.1%	36.8%	39.6%	42.1%	<0.001	27.0%	19.6%	21.5%	22.9%	24.8%	<0.001
Systolic blood pressure (mmHg)	122.8±13.6	122.9±13.2	124.5±13.4	125.8±13.7	127.1±14.1	<0.001	119.7±15.3	116.0±14.0	117.3±14.2	118.1±14.5	118.7±14.8	<0.001
Diastolic blood pressure (mmHg)	76.7±9.4	77.0±9.3	78.2±9.5	79.1±9.6	79.9±9.8	<0.001	74.1±9.9	72.7±9.6	73.8±9.8	74.4±10.0	74.8±10.2	<0.001
Fasting serum glucose (mg/dL)	97.6±23.6	96.2±21.2	97.7±22.3	99.4±23.7	101.7±26.7	<0.001	94.8±19.4	92.0±16.0	92.9±16.6	93.6±17.7	94.4±18.8	<0.001
Serum creatinine (mg/dL)	1.00±0.16	1.00±0.15	1.00±0.16	0.99±0.16	0.98±0.16	<0.001	0.76±0.13	0.77±0.13	0.77±0.14	0.76±0.14	0.76±0.14	<0.001
Serum total cholesterol (mg/dL)	191.8±35.9	192.5±35.0	194.6±35.3	196.0±35.7	195.9±36.6	<0.001	197.1±37.4	190.0±35.1	189.7±35.1	189.9±35.5	190.1±36.3	<0.001
Serum HDL cholesterol (mg/dL)	50.0±12.0	52.0±12.3	53.5±12.7	54.7±13.2	55.9±13.9	<0.001	57.3±13.4	61.6±13.9	64.3±14.7	65.5±15.2	66.4±16.0	<0.001
Serum LDL cholesterol (mg/dL)	114.6±33.8	113.0±33.0	111.1±33.8	108.9±34.8	105.5±36.2	<0.001	117.3±34.6	109.4±32.6	105.4±32.8	102.9±33.7	100.6±34.4	<0.001
Serum triglycerides (mg/dL)	136.5±85.6	138.3±89.8	151.6±100.4	163.5±110.0	174.9±121.6	<0.001	111.5±68.0	94.0±58.2	99.9±64.2	107.0±71.5	115.9±80.9	<0.001
eGFR (mL/min/1.73 m^2^)	89.8±15.5	92.2±15.6	92.7±15.4	92.9±15.3	92.9±15.2	<0.001	91.2±16.2	96.5±17.1	97.6±17.2	98.3±17.3	98.3±17.4	<0.001

Abbreviations: DM, diabetes; HT, hypertension; HDL, high-density lipoprotein; LDL, low-density lipoprotein; eGFR, estimated glomerular filtration rate by the Chronic Kidney Disease Epidemiology Collaboration equation based on serum creatinine; Regular exercise: moderate and/or vigorous intensity exercise 3 times or more per week. Mean ± standard deviation; Jonckheere-Terpstra test for trend in the response with numerical variables and with Cochran-Armitage test for trend in the response with categorical variables.

### Association between alcohol intake and incident proteinuria

**Fig 2:**
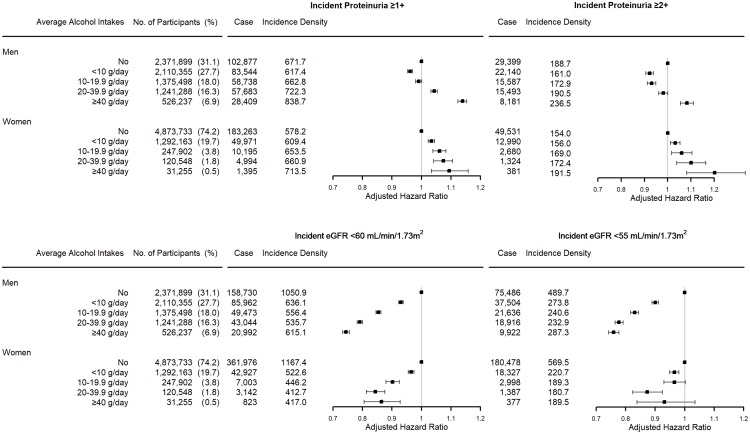
The association between alcohol intake and the risk of incident proteinuria and low estimated glomerular filtration rate (eGFR). Incident proteinuria was defined as ≥1+ or 2+ spot urine dipstick test for proteinuria in the participants who showed negative dipstick test at baseline. Incident low eGFR was defined as a decrease in eGFR <60 or 55 mL/min per 1.73 m^2^ in the participants who had eGFR ≥60 mL/min per 1.73 m^2^ at baseline. eGFR was calculated with the Chronic Kidney Disease Epidemiology Collaboration equation based on serum creatinine level. The risk was analyzed with an extended Cox model with alcohol intake level as a time-varying determinant, adjusted for baseline characteristics; age, sex, body mass index, systolic blood pressure, fasting serum glucose, serum high-density lipoprotein cholesterol and triglycerides levels, history of diabetes and hypertension medication, regular exercise, smoking status, and eGFR for incident proteinuria. Dots and error bars represent the adjusted hazard ratio and 95% confidence interval, respectively.

**Fig 3:**
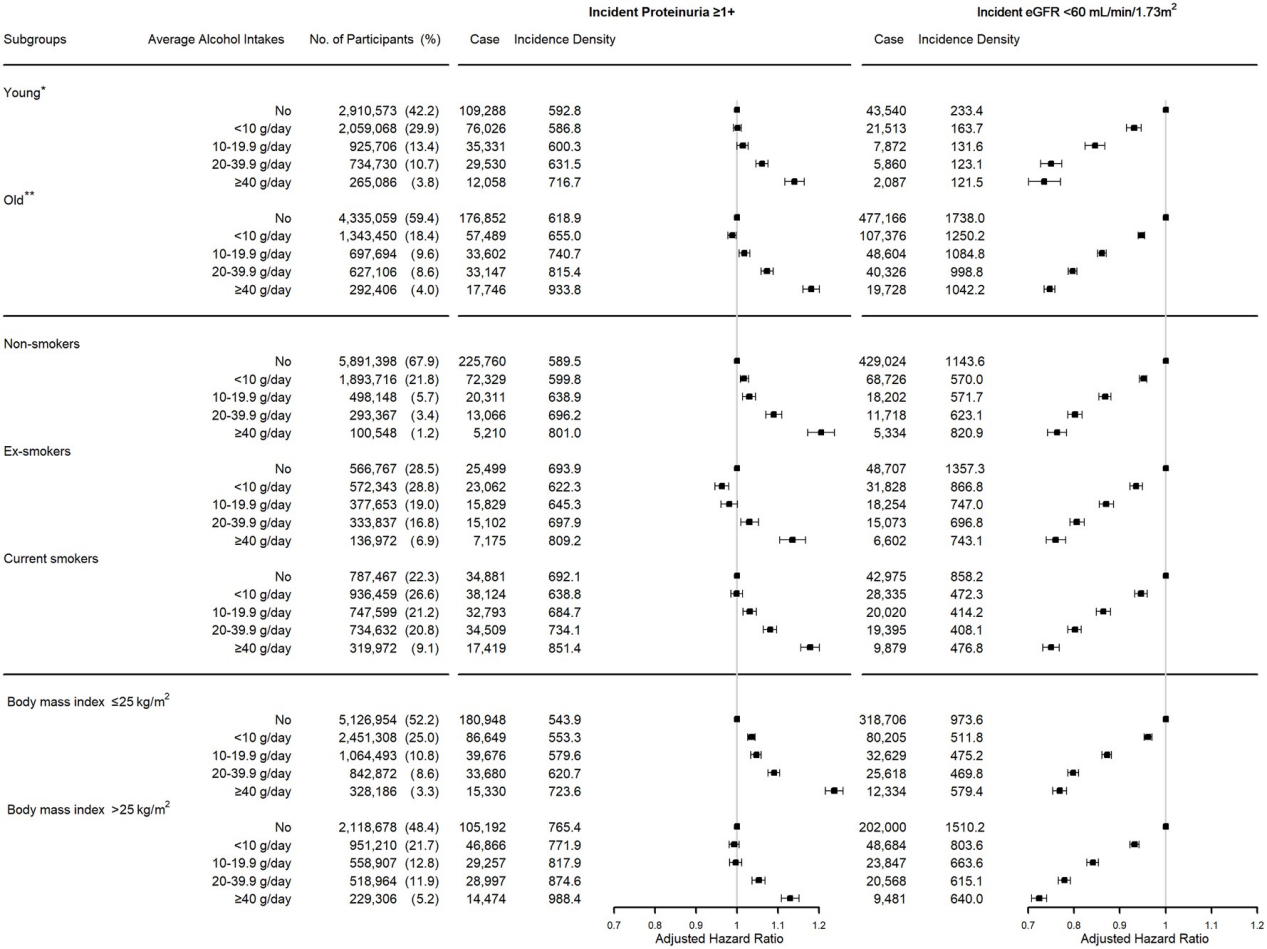
The association between alcohol intake and the risk of incident proteinuria and low estimated glomerular filtration rate (eGFR) in various subgroups. The old group was older than the sex-specific median age (44 years in men and 48 years in women). Incident proteinuria was defined as 1+ or higher spot urine dipstick test for proteinuria in the participants who showed a negative dipstick test at baseline. Incident low eGFR was defined as a decrease in eGFR <60 mL/min per 1.73 m^2^ in the participants who had eGFR ≥60 mL/min per 1.73 m^2^ at baseline. eGFR was calculated with the Chronic Kidney Disease Epidemiology Collaboration equation based on serum creatinine level. The risk was analyzed with an extended Cox model with alcohol intake level as a time-varying determinant, adjusted for baseline characteristics; age, sex, body mass index, systolic blood pressure, fasting serum glucose, serum high-density lipoprotein cholesterol and triglycerides levels, history of diabetes and hypertension medication, regular exercise, smoking status, and eGFR for incident proteinuria. Dots and error bars represent the adjusted hazard ratio and 95% confidence interval, respectively.

Incident proteinuria developed in 331,251 men and 249,818 women during 49,084,272.7 person-years in men (674.9 per 100,000 person-years) and 42,405,387.7 person-years in women (589.1 per 100,000 person-years). Alcohol ingestion <10 g/day showed a protective effect on the development of incident proteinuria in men, but this protective effect was not observed in women. The adjusted hazard ratio (aHR) for incident proteinuria was 0.961 (95% confidence interval [CI], 0.953–0.970) in men drinking <10 g/day, 0.990 (95% CI, 0.980–1.001) in men drinking between 10 and 19.9 g/day, 1.043 (95% CI, 1.032–1.054) in men drinking between 20 and 39.9 g/day, and 1.139 (95% CI, 1.123–1.154) in men drinking ≥40 g/day, compared with non-drinking men. The aHR for incident proteinuria was 1.034 (95% CI, 1.023–1.044) in women drinking <10 g/day, 1.061 (95% CI, 1.039–1.083) in women drinking between 10 and 19.9 g/day, 1.074 (95% CI, 1.042–1.106) in women drinking between 20 and 39.9 g/day, and 1.094 (95% CI, 1.034–1.158) in women drinking ≥40 g/day, compared with non-drinking women (Fig 2). The beneficial effect of modest alcohol ingestion was observed only in an old population who were older than the sex-specific median age and ex-smokers (Fig 3). When incident proteinuria was defined as a urine dipstick test for proteinuria ≥2+, the protective effect of alcohol intake was observed in the participants who drank <20 g/day, and the risk of those who drank ≥40 g/day was higher than that of non-drinkers in men. In women, the definition of incident proteinuria as ≥2+ dipstick test for proteinuria did not substantially change the results (Fig 2).

### Association between alcohol intake and change of eGFR

**Fig 4:**
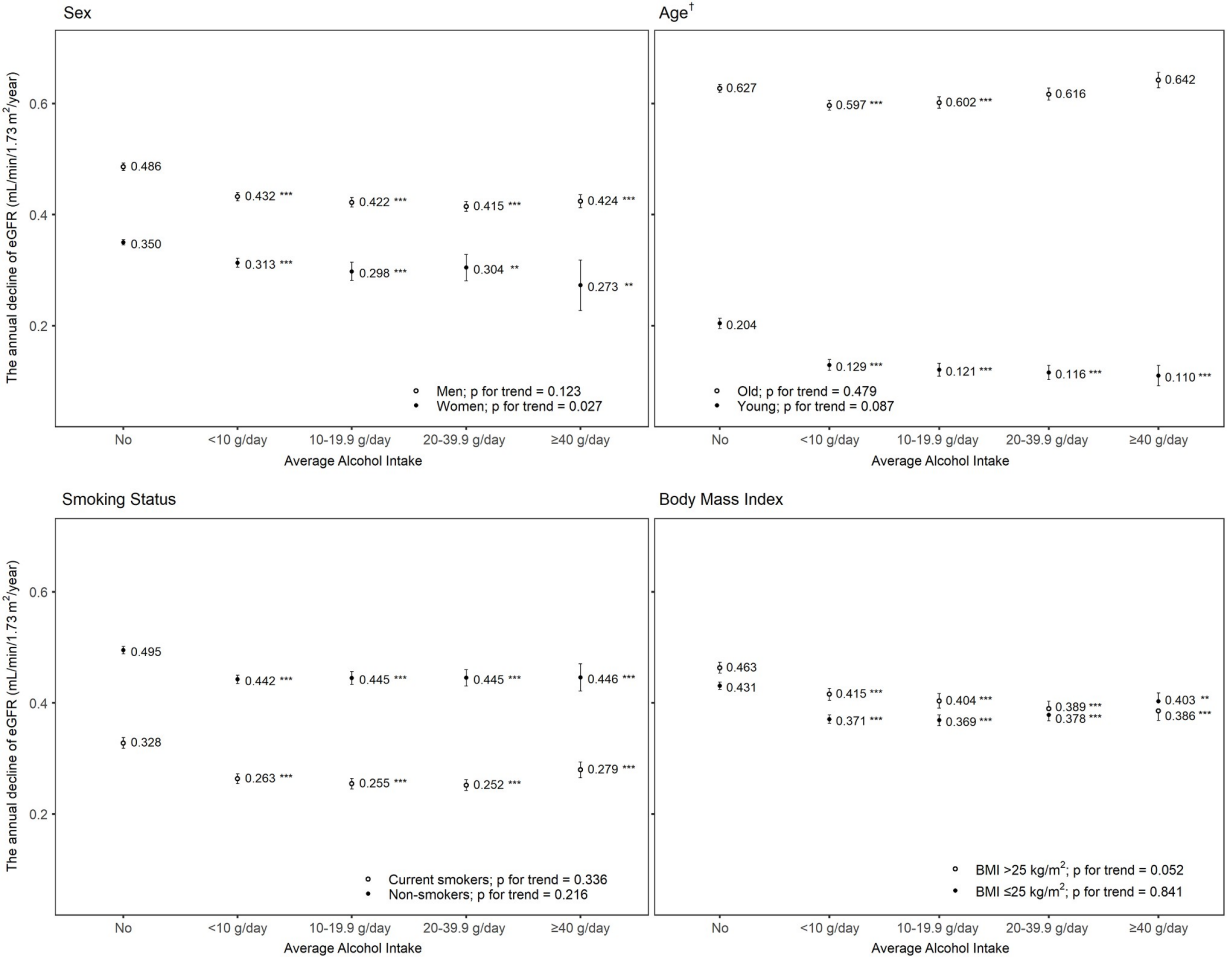
The association between alcohol intake and the annual decline of estimated glomerular filtration rate (eGFR). The estimated mean of decline change in eGFR was calculated with a generalized linear model adjusted for baseline characteristics; age, sex, body mass index, systolic blood pressure, fasting serum glucose, serum high-density lipoprotein cholesterol and triglycerides levels, history of diabetes and hypertension medication, regular exercise, and smoking status. Dots and error bars represent the estimated mean of the annual decline in eGFR and 95% confidence interval, respectively. * *p*<0.05 compared with non-drinkers, ** *p*<0.01, *** *p*<0.001, and † old group older than the sex-specific median age (44 years in men and 48 years in women).

In both men and women, alcohol intake was associated with a smaller annual decline of eGFR. The estimated mean annual decline of eGFR was 0.486 mL/min/1.73 m^2^ per year (95% CI, 0.479–0.493) in non-drinking men, 0.432 (95% CI, 0.425–0.440) in men drinking <10 g/day, 0.422 (95% CI, 0.413–0.431) in men drinking between 10 and 19.9 g/day, 0.415 (95% CI, 0.405–0.424) in men drinking between 20 and 39.9 g/day, and 0.424 (95% CI, 0.413–0.436) in men drinking ≥40 g/day (*p* for trend 0.206). The estimated mean annual decline of eGFR was 0.350 mL/min/1.73 m^2^ per year (95% CI, 0.345–0.355) in non-drinking women, 0.313 (95% CI, 0.305–0.321) in women drinking <10 g/day, 0.298 (95% CI, 0.281–0.314) in women drinking between 10 and 19.9 g/day, 0.304 (95% CI, 0.281–0.328) in women drinking between 20 and 39.9 g/day, and 0.273 (95% CI, 0.227–0.318) in women drinking ≥40 g/day (*p* for trend 0.027; Fig 4). Although this inverse association was observed regardless of smoking and BMI, a J-shape association was observed in the old age group. The annual decline in eGFR in the old age group was lower in the participants who drank <10 g/day (estimated mean 0.597; 95% CI, 0.588–0.605; *p*<0.001) and higher in those who drank ≥40 g/day (0.642, 0.628–0.656), compared with non-drinkers (0.627, 0.620–0.634; Fig 4), although the difference between non-drinking old age group and those who drank ≥40 g/day was not statistically significant.

Incident low eGFR was developed in 358,201 men and 415,871 women during 48,957,546.0 person-years in men (731.7 per 100,000 person-years) and 41,748,806.8 person-years in women (996.1 per 100,000 person-years). The risk of incident low eGFR was inversely associated with the amount of alcohol intake. The aHR of incident low eGFR was 0.930 (95% CI, 0.923–0.938) in men drinking <10 g/day, 0.854 (95% CI, 0.846–0.863) in men drinking between 10 and 19.9 g/day, 0.791 (95% CI, 0.782–0.800) in men drinking between 20 and 39.9 g/day, and 0.744 (95% CI 0.733–0.756) in men drinking ≥40 g/day, compared with non-drinking men. The aHR of incident low eGFR was 0.965 (95% CI, 0.955–0.975) in women drinking <10 g/day, 0.902 (95% CI, 0.880–0.925) in women drinking between 10 and 19.9 g/day, 0.844 (95% CI, 0.813–0.876) in women drinking between 20 and 39.9 g/day, and 0.864 (95% CI, 0.805–0.928) in women drinking ≥40 g/day, compared with non-drinking women (Fig 2). This inverse association was observed regardless of age, smoking status, and BMI status (Fig 3). The definition of low eGFR as <55 mL/min per 1.73 m^2^ did not substantially change the results (Fig 2).

## Discussion

In the analysis of health screening data of more than 14 million out of 36 million adults in Korea who underwent health screening ≥3 times between 2009 and 2016 and had negative dipstick test for urine protein and eGFR ≥60 mL/min per 1.73 m^2^ at baseline, we observed a J-shaped association between alcohol intake and incident proteinuria risk in men. The lowest risk was seen in men who drank <10 g/day, and a positive association was seen without beneficial effect in women. We also observed an inverse association between alcohol intake and annual decline in eGFR and incident low eGFR risk in both sexes.

Although the observations of this study were mostly consistent with a Japanese study that observed a beneficial effect of moderate alcohol intake (<20 g/day) on stage I or II CKD and an inverse association between alcohol intake and stage III CKD after 10-year follow-up of 123,764 middle-aged adults [16], the previous observations on the association between alcohol intake and the measures of CKD have been very contradictory.

Koning et al reported an inverse association between alcohol consumption and incident CKD after 10.2 years of follow-up [4], but they did not separately analyze low eGFR and proteinuria. An inverse association between alcohol intake and low eGFR was observed in a cross-sectional study of Taiwanese men [5]. An inverse association [6, 7], positive association [8], and a J-shaped association [9] between alcohol intake and incident low eGFR has been reported in longitudinal studies in the general population. A beneficial effect of moderate alcohol consumption has been reported in patients with immunoglobulin A nephropathy [10].

The association between alcohol intake and incident proteinuria/albuminuria has not been studied well enough. A cross-sectional study observed an increased prevalence of albuminuria in heavy drinkers who drank >32 g ethanol/day [11]. In a cross-sectional study performed in Japan, a beneficial effect of modest alcohol intake on the prevalent low eGFR <60 mL/min per 1.73 m^2^ was observed in both smokers and non-smokers but the beneficial effect on prevalent proteinuria was observed only in non-smokers [12]. A positive association between alcohol intake and incident proteinuria has been reported [7]. A J-shaped association between alcohol intake and incident proteinuria has been reported in Japanese men [13], and an inverse relationship between alcohol intake and incident end-stage renal failure in middle-aged Chinese men [20] was reported.

The inconsistency between the studies may be due to many reasons, including but not limited to inconsistent definitions of CKD measures, inconsistent categorization of the levels of alcohol intake, variations in follow-up duration, and variations in the characteristics of the study populations such as ethnicity, sex ratio, and modest sample size. Although eGFR was evaluated with modification of diet in renal disease equation in most previous studies, Koning et al evaluated with cystatin and creatinine-based method [4], Schaffner et al with Cockcroft-Gault equation [6], and Sato et al and Uehara et al with Japanese equation [9, 13]. The thresholds of low eGFR were variable, too. Mostly it was 60 mL/min per 1.73 m^2^ but Schaeffner et al defined with 55 mL/min per 1.73 m^2^ [6] and Koning et al with 90 mL/min per 1.73 m^2^ [4]. Proteinuria was defined as 1+ or higher by single urine dipstick test by Yamagata et al [16], as 1+ or higher by two consecutive tests by Uehara et al [13], by 24 hour-urine albumin amount by Koning et al [4], and by spot urine albumin-to-creatinine ratio by White et al [7]. The classification of alcohol ingestion extent was also variable. The maximal level of alcohol ingestion was 20 g/day in the studies reported by Yamagata et al [16], 30 g/day in the study reported by White et al and Koning et al [4,7], and 70 g/day in the studies reported by Sato et al and Uehara et al [9, 13]. The maximal level of alcohol ingestion of some studies seemed to be too low to represent the overall drinking amount of general population [4,7,16].

The samples of the previous studies were mostly less than ten thousand participants. Yamagata et al reported observations from more than 100 thousand participants and observed a J-shaped association between alcohol ingestion and the risk of CKD [16]. The participants of the study reported by Yamagata et al, were older than 40 years and those of our study were older than 20 years. In their study, the level of alcohol ingestion was classified as none, occasional, <20 g/day, and >20 g/day, and daily alcohol ingestion of 20 g seemed to be too low as the cut-off value of maximal level of alcohol ingestion to evaluated the harmful effect of high alcohol ingestion. The proportion of the participants who drank >20 g/day of alcohol was 6.2% in men and 0.1% in women, which was much lower than other previous reports, might not represent the drinking habits of general population. The participants of their study were annual health check-ups and signed informed consents and might be highly selective subjects with relatively healthy lifestyles, not representative of general population. Therefore, it is necessary to study the association between alcohol intake and the risk of CKD in samples that can better represent the general population. Considering the modest impacts of lifestyle factors on health outcomes and complexity between various lifestyle factors, a study population covering a significant proportion of the entire population may be one of the most important prerequisites of the study on the association between lifestyle factors and health outcomes. The huge size of our study population covering almost 40% of the whole adult population in Korea and the compulsory nature of the health screening of our study which made the subjects who might less likely be interested in their health and lifestyle habits, be participated in this study, might make our observations more robust based on the range of alcohol intake of the general population and the statistical power.

Most previous studies analyzed the association between alcohol ingestion and the risk of incident CKD defined as eGFR less than a threshold level in the participants whose baseline eGFR was above the threshold and did not analyze the association between alcohol ingestion and the annual change of eGFR which could more precisely indicate the nature of the association. In our study, an inverse association between the annual change of eGFR according to the level of alcohol ingestion was also observed.

Because daily alcohol intake was associated with an increase in blood pressure, a reduction in alcohol intake reduced blood pressure [21, 22], and high blood pressure is one of the most important risk factors for CKD, the negative association between alcohol intake and the risk of incident low eGFR and the beneficial effect of moderate alcohol intake on the development of incident proteinuria in men is intriguing. The exact mechanism(s) of the protective effect of moderate alcohol intake in the risk of proteinuria is not clear. Regular drinking produced an antiartherogenic lipoprotein profile such as elevated high-density lipoprotein-2/high-density lipoprotein-3 ratio and apolipoprotein A-1/apolipoprotein B ratio, compared with irregular drinking in squirrel monkeys [23]. In another animal study, a low concentration of ethanol protected podocytes through acetaldehyde dehydrogenase and 20-hydroxyeicosatetraenoic acid [24]. In human studies, various mechanisms such as serum high-density lipoprotein cholesterol, fibrinolytic activity, and insulin sensitivity have been suggested [25–28]. From autopsy data, an inverse correlation between alcohol consumption and hyalinization of renal arterioles was observed [29]. Overestimation of the true GFR using the serum creatinine-based estimation equation has been suggested, although the extent to which this factor affected the accurate measurement of true GFR in the general population is unknown [7].

The inverse association between alcohol intake and annual eGFR decline should be interpreted cautiously because increased eGFR defined by muscle mass adjusted criteria has been associated with increased all-cause mortality in the general population [30] and the implication of this inverse association needs to be evaluated with future longitudinal studies with a long enough follow-up period.

In this study, the beneficial effect of modest alcohol intake on incident proteinuria was observed only in men. The explanations for the observed sex differences in the pattern of association between alcohol ingestion and the risk of incident proteinuria are not clear. Differences in body composition and physiologic response might cause more severe consequences in women compared with men, even within a similar level of drinking [31]. The qualitative and quantitative difference according to sex in response to alcohol exposure deserves future studies.

This study has some limitations. First, the data were collected from many health screening centers across the country, and the protocols were not strictly standardized although standardized protocols are mandatory, such as questionnaires. Second, the proteinuria was evaluated with a dipstick test rather than using the urine albumin-to-creatinine ratio. Although the urine albumin-to-creatinine ratio is preferred over the dipstick test nowadays, the urine dipstick test has shown comparable results in many cohort studies [32]. Third, GFR was not measured but rather estimated using the Chronic Kidney Disease Epidemiology Collaboration equation based on serum creatinine concentration. Measurement of GFR is not practical for studies with large populations. Fourth, although health screening in Korea is mandatory, there is a possibility of underrepresentation of vulnerable groups. The participants in this study had normal eGFR and negative urine dipstick test for proteinuria, and the possibility is quite low that underrepresentation of vulnerable groups caused biased results. Fifth, lifetime abstainers could not be differentiated from past drinkers in our study, because there was no question regarding to this issue in the questionnaire. The difference in the risk of incident CKD between these two groups might explain the J-shape association between the alcohol ingestion level and the risk of incident proteinuria. Last, almost all of the study population in this study was ethnic Koreans, and caution should be taken when making generalizations based on the observations of this study.

Despite of the limitations, this study has merits. To the best of our knowledge, this study is the largest longitudinal study among those on the association between alcohol intake and measures of incident CKD. This study analyzed data collected before the development of measures of CKD and was free of various pitfalls of retrospective studies, such as recall bias. The huge size of this study allowed us to analyze results separately according to sex and various subgroups, and a beneficial effect of modest alcohol intake on incident proteinuria was observed only in men, the older age group, and ex-smokers. The etiology of CKD in the elderly population is known to be different from that in the younger population. In a Spanish series, albuminuria in subjects older than 65 years was more frequently associated with diabetes and hypertension compared with the younger subjects [33], and improved insulin sensitivity due to moderate alcohol intake [27] may be associated with a beneficial effect on proteinuria risk in the old group. The inverse association of alcohol intake with eGFR decline or incident low eGFR was not different according to sex, but an inverse association of alcohol intake with eGFR decline was observed in the young group and a J-shape association was seen in the old group. This differential association according to age may be explained by the difference in CKD etiology between age groups [33]. The possible differential association between modest alcohol intake and incident proteinuria according to age and smoking status needs to be confirmed with future studies. Besides the huge size of the study population, we analyzed data from the participants with at least three serum creatinine values at least 6 months apart, which must have reduced the bias caused by the variation in serum creatinine values due to nonrenal causes such as diet [34].

In conclusion, in this study consisting of about 40% of the entire adult population in Korea, we observed an inverse association between alcohol intake and eGFR decline and incident low eGFR in both sexes and a beneficial effect of modest alcohol intake on the risk of incident proteinuria in men. However, alcohol intake of any level was associated with an increased risk of incident proteinuria in women. The long-term implications of the association between alcohol and CKD measures should be elucidated with future studies.
